# Fibrosing mediastinitis in a child with Mendelian susceptibility to mycobacterial disease possibly due to *Bacillus* Calmette-Guérin

**DOI:** 10.1186/s13223-022-00738-3

**Published:** 2022-11-17

**Authors:** Niusha Sharifinejad, Seyed Alireza Mahdaviani, Shahrzad Fallah, Nasrin Khakbazan Fard, Alireza Norouzi, Mahnaz Jamee, Mahnaz Sadeghi-Shabestari, Majid Marjani, Mehran Malekshoar, Parisa Farnia, Ali Akbar Velayati

**Affiliations:** 1 Non-Communicable Diseases Research Center, Alborz University of Medical Sciences, Karaj, Iran; 2 Pediatric Respiratory Diseases Research Center, National Research Institute of Tuberculosis and Lung Diseases (NRITLD), Shahid Beheshti University of Medical Sciences, Tehran, Iran; 3 Pediatric Infectious Research Center, Mofid Children’s Hospital, Shahid Beheshti University of Medical Sciences, Tehran, Iran; 4 Pediatric Nephrology Research Center, Research Institute for Children’s Health, Shahid Beheshti University of Medical Sciences, Tehran, Iran; 5 Division of Pediatric Immunology and Allergy, Children’s Hospital, Tabriz University of Medical Sciences, Tabriz, Iran; 6 Clinical Tuberculosis and Epidemiology Research Center, National Research Institute of Tuberculosis and Lung Diseases (NRITLD), Shahid Beheshti University of Medical Sciences, Tehran, Iran; 7 Shahid Beheshti University of Medical Sciences, Tehran, Iran; 8 Mycobacteriology Research Centre (MRC), National Research Institute of Tuberculosis and Lung Diseases (NRITLD), Shahid Beheshti University of Medical Sciences, Tehran, Iran

**Keywords:** Mendelian susceptibility to mycobacterial disease, MSMD, Fibrosing mediastinitis, FM, *Bacillus* Calmette-Guérin, BCG

## Abstract

**Background:**

Mendelian susceptibility to mycobacterial disease (MSMD) is an uncommon disorder with increased susceptibility to less virulent mycobacteria including *bacillus* Calmette-Guérin (BCG). Fibrosing mediastinitis (FM) is also a rare condition defined by excessive fibrotic reactions in the mediastinum. So far, some infectious organisms and autoimmune diseases have been introduced as possible etiologies of FM. However, no study has ever discussed the possible association of BCG infection and FM.

**Case presentation:**

In this study, we report a 3-year-old female presenting with persistent fever, weakness, and bloody diarrhea in addition to mediastinal lymphadenopathy, hepatosplenomegaly, and pleural and pericardial effusion. Further examinations established a diagnosis of MSMD based on her clinical condition, immunologic data, positive tests for mycobacterial species, positive family history, and genetic study (*IL12RB1* gene, c.G1193C, p.W398S). A year and a half later, she was referred with submandibular lymphadenitis and underwent immunologic work-up which revealed high inflammatory indices, a slight reduction in numbers of CD3 + and CD4 + cells as well as elevated CD16/56 + cell count and hyperimmunoglobulinemia. Purified protein derivative (PPD), QuantiFERON, and gastric washing test were all negative. Her chest computed tomography (CT) scan revealed suspicious para-aortic soft tissue and her echocardiography was indicative of strictures in superior vena cava and pulmonary veins. She further underwent chest CT angiography which confirmed FM development. Meanwhile, she has been treated with anti-mycobacterial agents and subcutaneous IFN-γ.

**Conclusion:**

In summary, we described a novel case of MSMD in a child presenting with granulomatous FM possibly following BCG infection. This is the first report introducing aberrant BCG infection as the underlying cause of FM. This result could assist physicians in identifying early-onset FM in suspicious cases with MSMD. However, more studies are required to support this matter.

## Introduction

Mendelian susceptibility to mycobacterial disease (MSMD) is an uncommon inborn error of immunity defined by increased susceptibility to less virulent mycobacteria including *bacillus* Calmette-Guérin (BCG) [1]. These patients predominantly manifest local or disseminated mycobacterial infection along with a higher chance of being infected by *Salmonella* spp*.*, *Listeria Monocytogenes*, and viruses, especially herpes virus family. The immunological work-up is generally normal among most MSMD patients [2]. Gene mutations involved in this disorder cause changes in the IFN-γ signaling pathway and alter the normal anti-mycobacterial defense [3]. Of those, biallelic *IL12RB1* mutation is the most frequent genetic defect that is found in about 60% of the patients diagnosed with MSMD [4].

Fibrosing mediastinitis (FM) is a rare condition described by an excessive fibrotic reaction in the mediastinum. Most patients with FM are young at the time of presentation and mainly develop symptoms related to obstruction or compression of mediastinal structures including superior vena cava, pulmonary veins, pulmonary arteries, the central airways, and the esophagus [5]. Although the etiology of FM is not fully understood, a causal relationship has been reported between infectious microorganisms such as *Histoplasma capsulatum* and *Aspergillus,* autoimmune diseases, and even some drugs with FM [6, 7]. FM has also been detected in patients infected by *Mycobacterium tuberculosis* with less prevalence in the childhood [8]. However, no study has ever discussed the possible association of BCG infection and FM.

Here, we studied a 3-year-old girl diagnosed with MSMD and carried a homozygous mutation in the *IL12RB1* gene (c.G1193C*,* p.W398S)*,* who later presented with lymphadenopathy due to BCG infection and FM.

## Methods

The patient’s demographic information and physical examinations were collected through her medical records or direct interview based on the diagnosis and management guidelines for primary immunodeficiency [9], after the patient and her parents took a written informed consent, as claimed by the principles of the ethics committee of Shahid Beheshti University of Medical Sciences.

The recorded laboratory data were: complete cell blood counts, T- and B-cells subsets (assessed using flow cytometry analysis), serum levels of immunoglobulins, gastric washing test, QuantiFERON and purified protein derivative (PPD) test (to examine mycobacterial infections). Other known causes of FM were excluded by history taking, physical examination, and laboratory results. Clinical diagnosis of MSMD has been determined according to criteria of the European Society for Immunodeficiencies [10]. Genomic DNA extraction was performed using the whole peripheral blood sample, followed by the whole-exome sequencing and confirmatory Sanger sequencing method according to a previously published pipeline [11].

## Case presentation

The patient is an Iranian girl born to consanguineous Azeri parents and had an older sister with similar symptoms and previous diagnosis of MSMD. Her sister presented with left axillary and supraclavicular lymphadenopathies within the first 2 months of life. Despite receiving anti-mycobacterial regimen and IFN-ɣ, her synptoms relapsed with abdominal lymphadenopathies and splenomegaly that got complicated with cutaneous fistulae leading to abdominal surgery and lymphadenectomy. She also experienced allergic rhinitis, septic arthritis, and pneumonia and was later diagnosed with MSMD according to her genetic evaluation.

Based on her positive family history of MSMD, our patient was recommended to avoid BCG vaccination, but, received this vaccine at birth. She was hospitalized at the age of 3 years old due to persistent fever, weakness, and bloody diarrhea. During her first admission hepatosplenomegaly was detected; furthermore, the chest computed tomography (CT) scan revealed pleural and pericardial effusion, mediastinal lymphadenopathy, and left lung collapse. Considering the situation, a pleural window was inserted for the patient, antibiotics with full coverage of gram negative and positive bacteria were prescribed, and she underwent pericardiocentesis. However, no microbial microorganism was isolated from the specimens and the patient was discharged after clinical improvement. Three months later and due to the persistence of her previous symptoms, she underwent gastric washing tests for probable mycobacterial infection. The results came back three times positive and BCG-osis was confirmed by polymerase chain reaction (PCR) identification of Mycobacterium bovis in the sputum. Therefore, she was treated with a combination of anti-mycobacterial drugs (isoniazid, rifampin, pyrazinamide, ethambutol) for 9 months. Meanwhile, based on the previous genetic evaluation of the patient and her family using whole-exon sequencing for possible defects of interferon gamma (IFN-γ) signaling pathway, the disgnosis of MSMD was established. Sequencing results confirmed a homozygous missence mutation of the *IL12RB1* gene (NM_001290024; exon 11; c.G1193C) in the patient and her sister that led to the amino acid change of p.W398S. Both parents were heterozygous for this mutation.

**Fig. 1:**
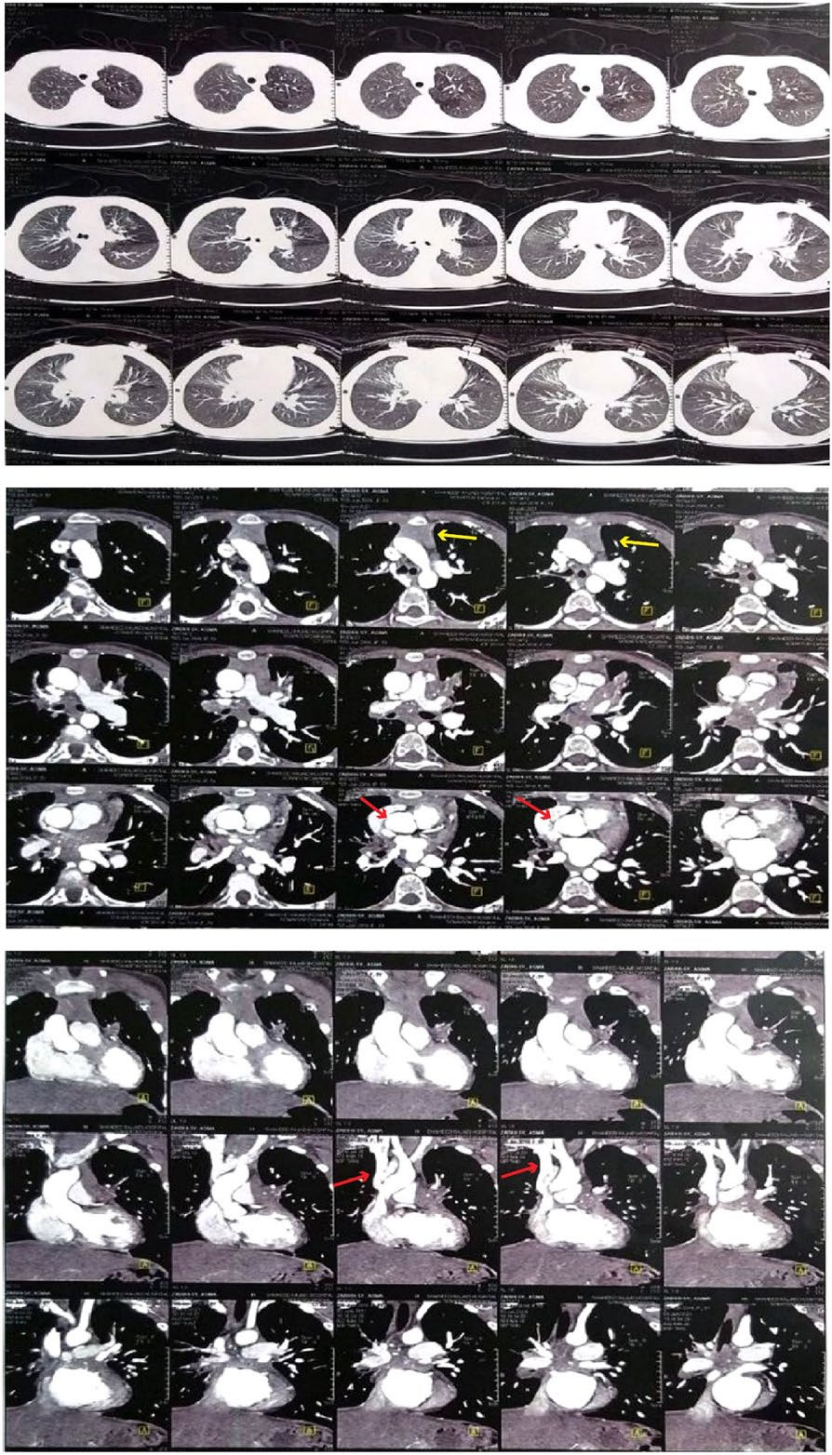
The chest CT angiography of the index patient. Narrowing in the distal part of superior vena cava (red arrows) and mediastinal fat infiltration (yellow arrows) with multiple calcifications

At the age of 4.5 years, she was referred to our hospital with submandibular lymphadenitis. In her primary physical evaluation, a 2 × 3 cm submandibular lymph node was detected. Laboratory work-up showed high inflammatory indices (erythrocyte sedimentation rate: 61, C-reactive protein: 59) with a slight reduction in CD3^+^ and CD4^+^ cell counts, increased CD16^+^56^+^cell count, and hyperimmunoglobulinemia (Table 1). Tests for detecting tuberculosis were all negative in the absence of ongoing anti-mycobacterial regimen: PPD, QuantiFERON, gastric washing test, microscopy of sputum samples for acid-fast bacilli, PCR and culture of sputum samples. A cervical sonogram reported multiple enlarged lymph nodes with reactive non-echogenic centers. Consent for lymphnode excisional biopsy from the parents could not be obtained. Abdominal ultrasound revealed enlarged para-aortic lymph nodes without hepatosplenomegaly. Chest CT scan showed massive and extensive lymphadenopathy in addition to ground glass and reticular opacities in the middle and lower lobes and a suspicious para-aortic soft tissue. Echocardiography was also indicative of strictures in the superior vena cava and pulmonary veins. Therefore, CT angiography was recommended and the patient was discharged on treatment with isoniazid, rifampin, ethambutol, subcutaneous IFNγ, and vitamin B6. About a year later, when she was solely on isoniazid, her cervical lymphadenitis relapsed. However, all other paraclinics were normal and no other site of lymphadenopathy was detected, thus, she was once again treated with the combination of isoniazid, rifampin, and ethambutol, which dwindled the mass. In her latest follow-up at the age of 6 years and while she still had minimal cervical lymphadenopathy, she underwent chest CT angiography. The result revealed narrowing in the distal part of superior vena cava and mediastinal fat infiltration with multiple calcifications, suggestive of FM. Thus, an investigation for other causes of FM, especially tuberculosis, was performed without any positive findings. The CT angiography of the patient is available in Fig. 1. Finally, given our patient did not experience obstructive symptoms of FM, we managed the patient expectantly.

**Table 1:** The immunologic data of the index patient

Parameters	Patient	Normal ranges
WBC × 10^3^ (cell/uL)	11	5.5–15.5
Hemoglobin (g/dL)	9	10.5–14.5
MCV (fl)	73	75–87
Lymphocytes %	6.93	1.5–8.5
Neutrophils %	3.08	1.5–7.5
CD3^+^ T cells (% of lymphocytes)	52	59.7–77.6
CD4^+^ T cells (% of T cells)	30	31.1–47.4
CD8^+^ T cells (% of T cells)	18	16.0–26.9
CD19^+^ (% of lymphocytes)	20	12.9–29.2
CD16^+^56^+^ (% of lymphocytes)	25	4.7–16.2
IgG (mg/dL)	2400	839.87 ± 164.19
IgA (mg/dL)	555	68.98 ± 34.05
IgM (mg/dL)	348	121.79 ± 39.24
IgE (IU/mL)	6	< 100
ESR (mm/first hour)	61	< 17
CRP (mg/l)	59	< 14
T cell response to BCG	1.5	> 2
T cell response to Canavalin	2.1	> 2

*WBC* White blood cells, *MCV* Mean corpuscular volume, *Ig* Immunoglobulin, *ESR* Erythrocyte sedimentation rate, *CRP* C-reactive protein, *BCG* *Bacillus* Calmette-Guérin

## Discussion

Fibrosing mediastinitis is a rare complication results from the excessive fibrotic reaction within the mediastinum and is categorized into two forms; non-granulomatous and granulomatous [5]. Whilst granulomatous FM mainly accompanies chronic infections caused by *Histoplasma capsulatum*, *Mycobacterium tuberculosis,* and fungi [8], immune-based diseases such as IgG4-related autoimmune disorders lead to non-granulomatous FM [12]. Despite the rare overall occurrence of FM [8], the long interval estimated between infection and FM development (6 years) is probably accountable for the lesser prevalence of FM among children [13].

In this study, we reported a 6-year-old girl diagnosed with MSMD and presented with relapsing lymphadenopathy and FM. She had a homozygous missense mutation of the *IL12RB1* gene (NM_001290024; exon 11; c.G1193C) similar to her sibling. The patient was administered with routine anti-mycobacterial regimen in addition to adjuvant IFN-γ therapy that can restore macrophage function and provide a better control of BCG-osis in MSMD patients, particularly in patients with IL-12Rβ1 deficiency [14]. Despite the promising results [1], there is no general indication to hematopeotic stem cell transplantation in genetic defects of the *IL12RB1* [15, 16] and our case was not evaluated for transplantation. The pericardial and pleural effusion primarily detected in our patient could also be attributed to the early stages of FM. The radiographic pattern of localized mediastinal fibrosis with areas of calcification and presence of pleural disease was coordinated to granulomatous FM in our patient, therefore, an infectious etiology was anticipated. However, we could not find any known infectious causes of FM except for BCG infection that is commonly present in MSMD patients. Similar to other microbial agents triggering FM development [12, 13], the abnormal inflammation originated from chronic relapsing BCG infections could be the underlying factor in our MSMD patient. Of note, the estimated interval between infection and FM seems to be shorter in our case, that might result from the persistant subacute BCG infection since the first months of life in MSMD patients [17]. Furthermore, the developement of FM has not been previously reported in any other inborn errors of immunity. Elevated immunoglobulins level (IgG, IgA, IgM) observed in our patient was similar to some previously reported MSMD cases [18]. Despite the variability of FM treatments based on the type and extent of the disorder, steroids are currently the main therapeutic option [5]. Since the diagnosis of FM is time consuming and symtoms can be present years before diagnosis, physicians may need to look for FM in suspicious cases of MSMD.

## Conclusion

In summary, we described a novel case of MSMD in a child presenting with granulomatous FM possibly following BCG infection. This the first report introducing BCG in an MSMD patient as the underlying cause of FM. However, more studies are required to support this matter.

## Data Availability

All data generated or analyzed during this study are included in this published article.

## Abbreviations

MSMD: Mendelian susceptibility to mycobacterial disease
BCG: *Bacillus* Calmette-Guérin
FM: Fibrosing mediastinitis
PPD: Purified protein derivative
CT: Computed tomography
Ig: Immunoglobulin
IFN-γ: Interferon gamma
PCR: Polymerase chain reaction

## References

[1] Taur PD, Gowri V, Pandrowala AA, Iyengar VV, Chougule A, Golwala Z (2021). Clinical and molecular findings in Mendelian susceptibility to mycobacterial diseases: experience from India. Front Immunol.

[2] Das J, Banday A, Shandilya J, Sharma M, Vignesh P, Rawat A (2021). An updated review on Mendelian susceptibility to mycobacterial diseases – a silver jubilee celebration of its first genetic diagnosis. Exp Rev Clin Immunol.

[3] Bustamante J (2020). Mendelian susceptibility to mycobacterial disease: recent discoveries. Hum Genet.

[4] Rosain J, Oleaga-Quintas C, Deswarte C, Verdin H, Marot S, Syridou G (2018). A variety of alu-mediated copy number variations can underlie IL-12Rβ1 deficiency. J Clin Immunol.

[5] Steven E Weinberger JCH. Mediastinal granuloma and fibrosing mediastinitis. 2021 [cited 31.07.2021]. In: UpToDate [Internet]. Waltham, MA: UpToDate, [cited 31.07.2021]. Available from: https://www.uptodate.com/contents/mediastinal-granuloma-and-fibrosing-mediastinitis#:~:text=Mediastinal%20granuloma%20is%20characterized%20by

[6] Mole T, Glover J, Sheppard M (1995). Sclerosing mediastinitis: a report on 18 cases. Thorax.

[7] Allard-Chamard H, Alsufyani F, Kaneko N, Xing K, Perugino C, Mahajan VS (2021). CD4(+)CTLs in fibrosing mediastinitis linked to histoplasma capsulatum. J Immunol.

[8] Wu Z, Jarvis H, Howard LS, Wright C, Kon OM (2017). Post-tuberculous fibrosing mediastinitis: a review of the literature. BMJ Open Respir Res.

[9] Abolhassani H, Tavakol M, Chavoshzadeh Z, Mahdaviani SA, Momen T, Yazdani R (2019). National consensus on diagnosis and management guidelines for primary immunodeficiency. Immunol Genet J.

[10] Seidel MG, Kindle G, Gathmann B, Quinti I, Buckland M, van Montfrans J (2019). The European society for immunodeficiencies (ESID) registry working definitions for the clinical diagnosis of inborn errors of immunity. J Allergy Clin Immunol Pract.

[11] Fang M, Abolhassani H, Lim CK, Zhang J, Hammarström L (2016). Next generation sequencing data analysis in primary immunodeficiency disorders - future directions. J Clin Immunol.

[12] Peikert T, Shrestha B, Aubry MC, Colby TV, Ryu JH, Sekiguchi H (2012). Histopathologic overlap between fibrosing mediastinitis and IgG4-related disease. Int J Rheumatol.

[13] Goussard P, Gie RP, Janson J (2018). Lethal fibrosing mediastinitis in a child possibly due to mycobacterium tuberculosis. Pediatr Pulmonol.

[14] Ying W, Liu D, Dong X, Wang W, Hui X, Hou J (2019). Current status of the management of Mendelian susceptibility to mycobacterial disease in Mainland China. J Clin Immunol.

[15] Lee WI, Huang JL, Yeh KW, Jaing TH, Lin TY, Huang YC (2011). Immune defects in active mycobacterial diseases in patients with primary immunodeficiency diseases (PIDs). J Formos Med Assoc.

[16] Rosenberg EB, Kanner SP, Schwartzman RJ, Colsky J (1974). Systemic infection following BCG therapy. Arch Intern Med.

[17] Bustamante J, Boisson-Dupuis S, Abel L, Casanova J-L (2014). Mendelian susceptibility to mycobacterial disease: genetic, immunological, and clinical features of inborn errors of IFN-γ immunity. Semin Immunol.

[18] Sharifinejad N, Mahdaviani SA, Jamee M, Daneshmandi Z, Moniri A, Marjani M (2021). Leukocytoclastic vasculitis in patients with IL12B or IL12RB1 deficiency: case report and review of the literature. Pediatr Rheumatol.

